# Fatal drug use in the COVID-19 pandemic response: Changing trends in drug-involved deaths before and after stay-at-home orders in Louisiana

**DOI:** 10.3389/fpubh.2023.1117841

**Published:** 2023-04-11

**Authors:** Maxwell M. Leonhardt, John R. Spartz, Arti Shankar, Stephen A. Murphy

**Affiliations:** ^1^Environmental Health Sciences, Tulane University School of Public Health and Tropical Medicine, New Orleans, LA, United States; ^2^Department of Biostatistics and Data Science, Tulane University School of Public Health and Tropical Medicine, New Orleans, LA, United States

**Keywords:** disaster mental health, overdose death, COVID-19, opioid, fentanyl, stimulant, disaster response, Louisiana

## Abstract

The effect of disaster events on increasing drug-involved deaths has been clearly shown in previous literature. As the COVID-19 pandemic led to stay-at-home orders throughout the United States, there was a simultaneous spike in drug-involved deaths around the country. The landscape of a preexisting epidemic of drug-involved deaths in the United States is one which is not geographically homogenous. Given this unequal distribution of mortality, state-specific analysis of changing trends in drug use and drug-involved deaths is vital to inform both care for people who use drugs and local policy. An analysis of public health surveillance data from the state of Louisiana, both before and after the initial stay-at-home order of the COVID-19 pandemic, was used to determine the effect the pandemic may have had on the drug-involved deaths within this state. Using the linear regression analysis of total drug-involved deaths, as well as drug-specific subgroups, trends were measured based on quarterly (Qly) deaths. With the initial stay-at-home order as the change point, trends measured through quarter 1 (Q1) of 2020 were compared to trends measured from quarter 2 (Q2) of 2020 through quarter 3 (Q3) of 2021. The significantly increased rate of change in Qly drug-involved deaths, synthetic opioid-involved deaths, stimulant-involved deaths, and psychostimulant-involved deaths indicates a long-term change following the initial response to the COVID-19 pandemic. Changes in the delivery of mental health services, harm reduction services, medication for opioid use disorder (MOUD), treatment services, withdrawal management services, addiction counseling, shelters, housing, and food supplies further limited drug-involved prevention support, all of which were exacerbated by the new stress of living in a pandemic and economic uncertainty.

## Introduction

On 11 March 2020, a statewide public health emergency declaration was enacted due to the COVID-19 pandemic, followed quickly by a stay-at-home order on 22 March 2020 (1). The state of Louisiana, along with the rest of the world, entered a time of societal disruption, social isolation, psychological stress, and fear (2, 3). The effect of large-scale disaster events on relapse rates and overdose rates related to substance use has been clearly demonstrated through observational studies and analyses (4).

Analyses from various states, including Massachusetts and California, have demonstrated worsening rates of drug-involved substance use, resulting from the pandemic response and societal disruptions (5, 6). Considering the variability in the rates of drug-involved deaths and the type of drug-involved deaths that exist from state to state, there is a need for the improved understanding of the changing trends at state and regional levels (7).

Louisiana has been struggling with consistently increasing rates of drug-involved deaths higher than the national average since 2010 (8). In 2016, the age-adjusted overdose death rate in Louisiana was more than 10% higher than the national average (9). The factor that probably contributed to this increase in mortality rate was the high opioid prescription rates from 2012 to 2016 in Louisiana, which was ranked among the top 10 countries with the highest mortality rates in 2012 and ranked fifth among countries with the highest mortality rates in 2016 (9). One factor that showed potential to mitigate the impacts of drug-involved was the expansion of Medicaid in 2016 in Louisiana (10). Following the expansion of Medicaid, in 2017, the Louisiana state began to take some corrective actions toward the rising overdose deaths by limiting the supply of opioids given with first-time prescriptions, expanding access to naloxone *via* a standing order for any resident to pick up naloxone without a formal prescription, and legalizing syringe access programs among other initiatives (9). Even with these changes, in 2019, prior to the local disruptions from the pandemic, Louisiana's low number of opioid treatment programs per capita ranked it as the 46th among the 50 states of USA and Washington, DC (11), and in this same year, the Louisiana state had the 16th highest age-adjusted death rate in the country (12). Considering the past challenges in the state of Louisiana, the following analysis will demonstrate the growing and changing trends in drug-involved deaths in Louisiana and contribute to a better local understanding of this crisis.

## Methods

### Samples and measures

Data were obtained from the Louisiana Opioid Surveillance Initiative published by the Louisiana Department of Health (13). Eligible data were obtained *via* the website using the topic of “Death” and choosing an available “measure” to identify drug-involved deaths and drug-involved death subgroups, that is, opioid-involved deaths and heroin in Louisiana, respectively. The results were displayed for each group and downloaded. These data files were individual for each group and contained death data from each Louisiana parish, as well as death data as a state total, broken down by quarter starting from Q1 of 2016 to Q3 of 2021 (Q1: January–March; Q2: April–June; Q3: July–September; Q4: October–December). Since the stay-at-home order occurred at the end of March 2020, we considered Q2 of 2020 and subsequent quarters to be the COVID-19-affected time and all preceding quarters (from Q1 of 2016 to Q1 of 2020) to be pre-COVID-19 quarters. According to the Louisiana Health Department, death data were sourced from the Louisiana Electronic Event Registration System in the Bureau of Vital Records and Statistics in the Office of Public Health. Data for the state totals were used in place of parish data, as data from any parish with <5 deaths were suppressed for confidentiality. No exclusions were made based on other criteria.

### Analyses

To put the drug-involved death data into the context of the greater population threat, the mortality rate per 100,000 persons was calculated for drug-involved deaths and each subgroup for the year 2020 within the state population of Louisiana using census data from 2020 (U.S. Census Bureau, 2020). Linear regression models were calculated to determine linear trends for Qly drug-involved and subgroup deaths for the pre-COVID-19 response period (Q1 of 2016 through Q1 of 2020) and separately for the post-COVID-19 response period (Q2 of 2020 through Q3 of 2021). Subsequent slope coefficients from the linear regression models were compared using the one-way analysis of covariance to determine if changes in trends before and after the beginning of the COVID-19 pandemic stay-at-home order for total drug-involved deaths and all subgroups were significantly different.

Visual representations of the total deaths from Louisiana drug-involved death and subgroups were generated as a line graph as well as from all data available, starting Q1 of 2016 through Q3 of 2021. Heroin-involved deaths and synthetic opioid-involved deaths were represented as a percentage of the total opioid-involved deaths in Louisiana which was visually represented as a bar graph.

Analyses were performed with IBM SPSS. No additional review or approvals were required, as aggregated data were extracted from publicly available public health surveillance systems and reports.

## Results

**Figure 1:**
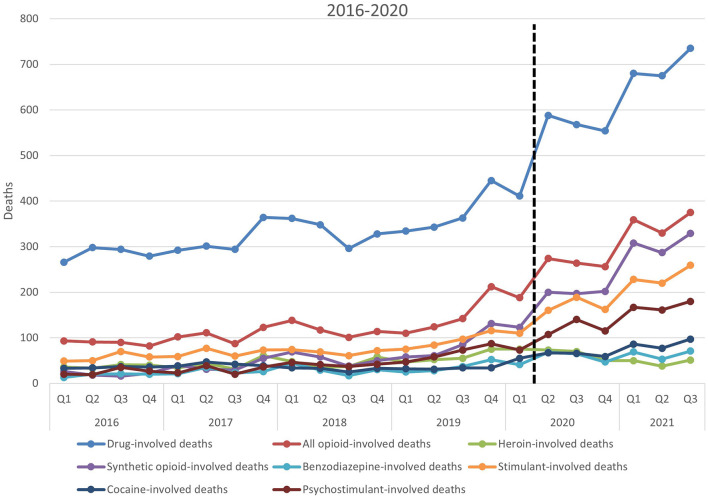
Total deaths of all drug-involved deaths and its subgroups in Louisiana State 2016–2020. Total deaths of all evaluated subgroups of death over the timeframe provided by these data. The dashed line marks the transition to data after the start of the COVID-19 pandemic; *y*-axis represents the total number of deaths; *x*-axis represents time in quarters by year.

A general positive trend in deaths in all drug-involved deaths and all subgroups is shown in Figure 1 from Q1 of 2016 to Q1 of 2020, with some variations in each quarter. After Q1 of 2020, which marks the last data prior to the onset of the COVID-19 pandemic, a sharp rise is noted from Q1 of 2020 to Q2 of 2020 by a strongly positive slope in line with that observed in drug-involved deaths and most subgroups. This is not the case for heroin-involved deaths (gray) which slopes downward or cocaine-involved deaths (dark blue) which stays relatively flat.

**Figure 2:**
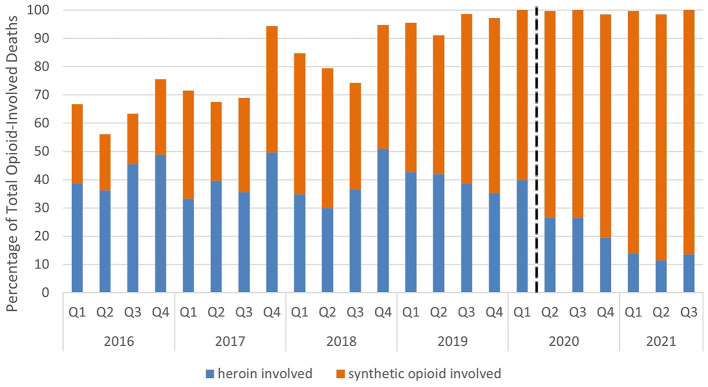
Heroin-involved and synthetic opioid-involved deaths as a percentage of total opioid-involved deaths. Heroin-involved deaths (blue) and synthetic opioid-involved deaths (orange) represented as a percentage of the total opioid-involved deaths from these data. The dashed line marks the transition to data after the start of the COVID-19 pandemic; *y*-axis represents the percentage of total opioid-involved deaths; *x*-axis represents time in quarters by year.

A trend of increasing synthetic opioid-involved deaths beginning in 2017 and becoming most apparent in 2019 is shown in Figure 2. The percentage of opioid-involved deaths involving heroin is decreasing as a trend, which is shown clearly in Figure 2. Most recent data from 2021 show synthetic opioid-involved deaths as a significant portion of opioid-involved deaths (from 85.8 to 87.7%).

An increase in mortality rate was observed for drug-involved and most defined subgroups between Q1 and Q2 in 2020, which was sustained for the rest of the year (Table 1). Drug-involved mortality increased steeply from Q1 to Q2 of 2020 (from 8.82 to 12.62 deaths per 100,000), dropped slightly in Q3 (12.19 deaths per 100,000), and continued to decrease into Q4 (11.89 deaths per 100,000) though never approaching Q1 of 2020 rates. All opioid-involved death rates followed a similar pattern with an increase from Q1 to Q2 of 2020 (from 4.04 to 5.88 deaths per 100,000) and decreasing between Q3 and Q4 of 2020 (5.67 and 5.50 deaths per 100,000, respectively). Benzodiazepine-involved deaths followed a similar pattern to all drug-involved mortality, increasing from Q1 to Q2 of 2020 (from 0.88 to 1.46 deaths per 100,000) and then declining between Q3 and Q4 of 2020 (between 1.40 and 1.01 deaths per 100,000, respectively). Cocaine-involved mortality followed a similar pattern, increasing from Q1 to Q2 of 2020 (from 1.18 to 1.44 deaths per 100,000) and slowly decreasing between Q3 and Q4 (from 1.42 to 1.27 deaths per 100,000, respectively). Synthetic opioid-involved mortality initially followed a similar pattern, increasing from Q1 to Q2 (from 2.64 to 4.29 deaths per 100,000, respectively) and a drop in Q3 (4.23 deaths per 100,000). However, notably, it is the only subgroup to increase from Q3 to Q4, reaching the highest rate it had in all quarters of 2020 (4.34 deaths per 100,000). Both stimulant- and psychostimulant-involved death rates increased from Q1 to Q2 and were the only subgroups to continue the rise in Q3 before decreasing in Q4 (stimulant-involved death rate was 2.36, 3.44, 4.06, and 3.48 deaths per 100,000 between Q1 and Q4, respectively; the psychostimulant-involved death rate was 1.57, 2.30, 3.01, and 2.47 deaths per 100,000 between Q1 to Q4, respectively). The heroin-involved death rate was the only subgroup to decrease from Q1 to Q2 (1.61 to 1.57 deaths per 100,000), and the rate continued to decrease to Q3 and Q4 (1.50 and 1.07 deaths per 100,000, respectively).

**Table 1:** Mortality rate of drug-involved deaths and subgroups in Louisiana during 2020.

**Group**	**Deaths per 100,000 individuals**			
	**Q1**	**Q2**	**Q3**	**Q4**
Drug-involved deaths	8.82	12.62	12.19	11.89
Opioid-involved deaths	4.04	5.88	5.67	5.50
Heroin-involved deaths	1.61	1.57	1.50	1.07
Synthetic opioid-involved deaths	2.64	4.29	4.23	4.34
Benzodiazepine-involved deaths	0.88	1.46	1.40	1.01
Stimulant-involved deaths	2.36	3.44	4.06	3.48
Cocaine-involved deaths	1.18	1.44	1.42	1.27
Psychostimulant-involved deaths	1.57	2.30	3.01	2.47

All drug-involved deaths and subgroups using data from the Louisiana Department of Health with mortality rate calculated using census data of the total population of Louisiana state (4,657,757) [5] and converted to deaths per 100,000 and rounded to the nearest hundredth decimal place.

The linear regression models (Table 2) revealed statistically significant positive linear trends for drug-involved deaths and for all subgroups, except for cocaine-involved deaths, in the period prior to the COVID-19 response (from Q1 of 2016 to Q1 of 2020). In the period following the stay-at-home COVID-19 response orders, linear regression analyses revealed statistically significant linear trends for drug-involved deaths and all subgroups, except benzodiazepine-involved deaths and cocaine-involved deaths. All the linear trends continued to be positive except the negative linear trend of heroin-involved deaths.

**Table 2:** Linear regression model coefficients of quarterly drug-involved and subgroup deaths in Louisiana for the quarter prior to and following the initial COVID-19 pandemic response and the univariate analysis of interaction.

	**Pre-COVID-19 response (Q1 of 2016–Q1 of 2020)**			**Post-COVID-19 response (Q2 of 2020–Q3 of 2021)**			**ANCOVA**
	*R* ^2^	**Slope (**β**)**	***p*** **-value**	*R* ^2^	**Slope (**β**)**	***p*** **-value**	***p*** **-value**
Drug-involved deaths	0.656	7.745	< 0.001^*^	0.743	33.771	0.027^*^	0.004^*^
Opioid-involved deaths	0.594	5.373	< 0.001^*^	0.695	23.029	0.039^*^	0.010^*^
Heroin-involved deaths	0.553	2.034	< 0.001^*^	0.671	−5.886	0.046^*^	0.003^*^
Synthetic opioid-involved deaths	0.733	5.711	< 0.001^*^	0.805	29.171	0.015^*^	< 0.001^*^
Benzodiazepine-involved deaths	0.466	1.412	0.003^*^	0.000	0.029	0.992	0.519
Stimulant-involved deaths	0.703	3.159	< 0.001^*^	0.786	18.686	0.019^*^	< 0.001^*^
Cocaine-involved deaths	0.002	0.066	0.851	0.624	6.000	0.061	0.005^*^
Psychostimulant-involved deaths	0.775	3.498	< 0.001^*^	0.759	13.714	0.024^*^	0.002^*^

Linear regression and ANCOVA calculated using SPSS.

* Significant when the *p*-value was < 0.05.

ANCOVA calculated for deaths per quarter with pre- and post-COVID-19 response as covariants.

The results from univariate analyses of covariance revealed significant differences between the pre- and post-pandemic response regression models (Table 2). Statistically significant differences in the models were identified for drug-involved deaths, heroin-involved deaths, opioid-involved deaths, synthetic opioid-involved deaths, stimulant-involved deaths, cocaine-involved deaths, and psychostimulant-involved deaths.

## Discussion

Agreeing with early reports on trends following the response to COVID-19, the rate of drug-involved death in Louisiana drastically and significantly increased following the initial societal disruptions resulting from the response to the COVID-19 pandemic, further worsening an already growing mortality risk in Louisiana (14). The nearly 6-fold increase in the slope of synthetic–opioid and stimulant-involved deaths and a 4-fold increase in the slope of psychostimulant-involved deaths, from pre-pandemic and extending beyond the initial stay-at-home order, notably signifies changes in trends of the types of drug-involved. These findings agree with reports from other jurisdictions indicating changes to the drug supply (15) and preferences in substance use (16, 17). Specifically, the change from a positive to a negative slope of heroin-involved deaths, revealing a reversal to the currently decreasing trend in heroin-involved deaths, points to potential changes in preference and/or availability of heroin in New Orleans, LA, USA.

Factors responsible for the identified changes in trends specific to the COVID-19 pandemic response on people who use drugs (PWUD) are multifaceted. Social isolation increased substance use in people with existing substance use disorder (SUD), initiated the use of substances to cope, and exacerbated psychological disorders such as anxiety, trauma-related stress disorder, or depression (18, 19). Unemployment and financial hardship worsened during the pandemic, thus driving increased substance use (3). The stay-at-home order reduced access to facilities essential to assisting those with substance use disorder (20, 21), facilities that housed harm reduction services, medication for opioid use disorder, treatment services, withdrawal management services, addiction counseling, mental health counseling, shelters, housing, and food supplies (20, 21). Societal disruptions and government mandates during the pandemic also resulted in changes to the global illicit drug supply, leading to an increase in toxic and adulterated compounds in circulation. Furthermore, longer periods of decreased drug availability likely led to an increased risk for individuals to experience withdrawal (21).

In the early stages of the pandemic response, federal, state, and local responses were elevated to prepare for the potential effects on PWUD. The Substance Abuse and Mental Health Services Administration (SAMHSA) and the United States Drug Enforcement Agency (DEA) quickly allowed doorstep delivery of MOUD and take-home doses of methadone for up to 28 days (22, 23). The DEA also waived the in-person medical evaluation as a requirement for prescribing buprenorphine and allowed off-site locations to dispense MOUD (22). The Centers for Disease Control and Prevention (CDC) issued guidance to syringe-service programs (SSPs) and comprehensive harm reduction agencies that include syringe-exchange programs (24). General instructions to prevent COVID-19 transmission, decrease unnecessary in-person contact, and offer larger quantities of syringes at each exchange were outlined. Louisiana state quickly adopted the federally issued allowances surrounding MOUD, recommended transition to telehealth services, and encouraged dispensation of the naloxone kits to everyone engaging with harm reduction services (25, 26). The United States and Louisiana had rapid, flexible responses to this novel pandemic as it unfolded. These preparations were largely influenced by the evaluation of previous natural disasters, noting the importance of cohesive emergency preparedness to allow for the continuity of operations of harm reduction services and substance use treatment services (3). The rapid response may have stymied the initial worsening trends, as evidenced by the improvements observed in most subgroups of drug-involved deaths through the second half of 2020, following the drastic increases observed in the first half of 2020. However, the continuation of worsening trends through the first three quarters of 2021, as evidenced by significant changes in slope for most of the subgroups, indicates a need for a revised focus on the risks for PWUD in Louisiana state and an appropriate response.

There are some limitations that should be considered when interpreting these findings. Non-fatal drug overdoses were not included in these data, which indicates the harmful impacts of COVID-19 due to changes in drug use patterns are likely greater than as demonstrated in this article. As drug overdose data were collected from death certificates, which were completed by elected coroners in each parish of the state of Louisiana, the data may be limited due to differences in each official's varied completion of death certificates. Drug-involved deaths may not be fully investigated leading to unspecified drug death categorization, further leading to underestimating the numbers of specific drug-involved death subgroups in the analysis. This is of particular interest in the state of Louisiana, as it has been previously identified that close to half of the overdose deaths in the state between 1999 and 2015 have been classified as unspecified instead of completely identified, leading to vast underestimations of, specifically, of opioid-involved deaths (27). The deaths with unspecified death certificates are, however, still captured within the category “drug-involved deaths” in this analysis. This is a limitation in understanding the full scale of the effects on different subgroups. There are also limitations inherent to the definition of drug-involved death and each death subgroup set forth by the Louisiana Department of Health (28). The temporality of COVID-19 was approximated as the end of Q1, and more refined dates of the various drug-involved deaths would provide a better understanding of the direct effect of the COVID-19 pandemic at different points of response. These limitations likely lead to a more conservative estimate of the relationship we observed.

## Conclusion

The 6 yearly quarters following the initial emergency declaration and stay-at-home orders issued in response to the COVID-19 pandemic in March 2020 led to the highest drug-involved mortality rates in the history of Louisiana State. The acute initial response at the federal, state, and local levels to counteract rising drug-involved deaths may have been effective after the first initial surge. However, the changes identified in drug use and overall deaths require awareness, flexibility, and investment in infrastructure to support PWUD. Furthermore, to create more situational awareness, standardized reporting procedures, and collection of overdose data to better inform policy and decision-making needs to be prioritized and fully supported across states and the federal government. The changing trends in drugs involved in these deaths as well as the rapid change in overall mortality demonstrate a gap in preparedness and require a reevaluation of how we collectively respond to future disasters and pandemics.

## Data availability statement

Publicly available datasets were analyzed in this study. This data can be found here: https://lodss.ldh.la.gov/.

## Author contributions

ML, JS, and SM contributed to the conception of the research. ML and JS implemented the research. ML performed the analyses and interpretation, as well as the writing of the manuscript. AS reviewed statistical analyses. SM advised and edited at all steps. All authors provided critical feedback and helped in shaping up the analyses, interpretation, and manuscript.
